# Tranilast enhances the anti-tumor effects of tamoxifen on human breast cancer cells *in vitro*

**DOI:** 10.1186/1423-0127-20-76

**Published:** 2013-10-21

**Authors:** Sara Darakhshan, Ali Ghanbari

**Affiliations:** 1 Department of biology, Faculty of science, Razi University, Kermanshah, Iran; 2 Fertility and Infertility Research Center, Kermanshah University of Medical Sciences, Kermanshah, Iran

**Keywords:** Breast cancer, Tamoxifen, Tranilast, Apoptosis, Transforming growth factor-beta

## Abstract

**Background:**

Tamoxifen is the most widely used anti-estrogen for the treatment of breast cancer. Studies show that the combination therapy with other substances that helps the activity of tamoxifen. The objective of this study was to evaluate the effect of tamoxifen when used in combination with tranilast on human breast cancer cells.

**Results:**

Two MCF-7 and MDA-MB-231 human breast cancer cell lines were treated with tamoxifen and/or tranilast. The cell viability and cytotoxicity was assessed using MTT and LDH assays; the apoptotic effects were examined by TUNEL assay, acridine orange/ethidium bromide staining and DNA laddering, also the expression levels of bax and bcl-2 genes were detected by real-time RT-PCR. The mRNA expression of TGF-β ligands and receptors examined using real-time RT-PCR and TGF-β1 protein secretion levels were also evaluated by ELISA assay. Inhibitory effect of these drugs on invasion and metastasis were tested by wound healing and matrigel invasion assay.

We found that combination of these drugs led to a marked increase in growth and proliferation inhibition compared to either agent alone. Furthermore, bax and bcl-2 affected by tamoxifen and/or tranilast and resulted in a significant increase in bax and decrease in bcl-2 mRNA expression. In addition, treatment with tamoxifen and/or tranilast resulted in significant decreased in TGF-β1, 2, 3, TGF-βRI and II mRNA and TGF-β1 protein levels while TGF-βRIII mRNA level was increased and invasion was also inhibited.

**Conclusions:**

These findings indicate that tranilast, by synergistic effect, enhances the activity of tamoxifen and the TGF-β pathway is a target for this combination therapy, therefore; we propose that this combined treatment may be suitable selection in prevention of breast cancer.

## Background

Apoptosis or programmed cell death provides an effective non-inflammatory way to remove redundant or damaged cells from tissues thereby acquiring tissue homeostasis [1]. Defective apoptosis and, in part, inappropriate proliferation, underpin the process of tumorigenesis [2] in addition, resistance to apoptosis is an important feature for cancer cells to invasion [3].

As estrogen significantly associated with the initiation, progression, even recurrence of breast cancer [4], anti-estrogens have important therapeutic potential in endocrine therapy for breast cancer. Tamoxifen ((Z)-1-{4-[2-(dimethylamino) ethoxy] phenyl}-1, 2-diphenyl-1-butene) (TAM) is a synthetic non-steroidal anti-estrogenic drug that widely used for the treatment or prevention of breast carcinoma [5]. Despite the relative safety and significant anti-neoplastic activities of tamoxifen, most initially responsive breast tumors develop resistance to its [6]. Even though an improved understanding, resistance to anti-estrogen therapy remains a significant clinical problem. However, combination therapies of tamoxifen with other drugs that aimed at the signaling pathways underlying the development of resistance may be a potential means of delaying the arrival of resistance.

One cytokine that may contribute to the metastatic potential and possibly tamoxifen resistance of tumor cells is transforming growth factor beta (TGF-β). There is three isoforms of TGF-β: TGF-β1,-β2 and -β3 [7]. Cell functions regulation by TGF-βs arises from his interaction with three discrete cell surface receptors, TGF-βRI, II and III [8]. TGF-β family regulates a diverse range of epithelial cell processes including proliferation, apoptosis, differentiation, adhesion and migration in a cell- and context-specific manner [9]. The multiplicity of TGF-β actions in nearly all cell types suggests that these have a complex and pivotal role in several physiological and pathological processes. TGF-β have an important role in normal mammary as a potent inhibitor of epithelial proliferation and regulator of mammary growth and development [10]. In addition, TGF-β plays complex roles in breast carcinogenesis. Early in mammary carcinogenesis the TGF-β signaling pathway functions as a tumor suppressor [11] however, at later stages, levels of TGF-β increase with tumor progression suggesting that TGF-β is now stimulating breast cancer progression [12].

The drug tranilast (N-[3′, 4′-dimethoxycinnamoyl]-anthranilic acid), an anti-allergic agent, has been applied for bronchial asthma, allergic rhinitis and atopic dermatitis, also suppresses collagen synthesis in keloid or hypertrophic scars [13,14]. The inhibitory effect of tranilast in different cell types is probably by antagonizing and inhibiting synthesis and secretion of TGF-β [15-19]. Since tranilast responsibilities through TGF-β pathway, it seems also tamoxifen influences this pathway [20], we hypothesize that combination of tamoxifen and tranilast may an appropriate therapeutic option for breast cancer management. In this paper, possible synergistic effect of tamoxifen with tranilast was examined in the hope of creating a more effective anti-tumor treatment strategy.

## Methods

### Cell lines & drugs

MCF-7 (noninvasive human breast adenocarsinoma, ER- and PR-positive) and MDA-MB-231 (metastatic human breast adenocarsinoma, ER-, PR- and HER2-negative) obtained from the National Cell bank of Iran (NCBI), were grown in RPMI-1640 media supplemented with 10% (v/v) fetal calf serum (FCS) and penicillin/streptomycin antibiotics. Cultures were maintained at 37°C in a humidified atmosphere of 5% CO_2_ in air. TAM and tranilast were purchased from Enzo Life Sciences and dissolved in dimethyl sulfoxide (DMSO) so that the final dimethyl sulfoxide concentration in experimental wells did not exceed 0.5% (v/v). Aliquots of a 1000 μM stock solution of TAM and tranilast were stored in dark at -70°C, defrosted and diluted with cell culture medium to the desired concentration before use.

The concentrations used alone treatment were the following: TAM: 1, 2, 5, 10 and 20 μM; tranilast: 10, 20, 50, 100 and 200 μM. The treatment combinations used were: 2 μM of TAM with different concentrations of tranilast: 10, 20, 50, 100, and 200 μM for 48 h.

### Cell viability measurement

Cytotoxic effect of TAM and tranilast was determined by MTT test. MCF-7 or MDA-MB-231 cells were seeded in 96-well culture plates at 10^4^ cells/well density. Cells were allowed to attach for 24 h before drugs were added to the medium. All drug concentrations were tested in triplicate wells and the assays were performed in three separate experiments. Following 48 h exposure at 37°C and 5% CO_2_, 20 μl MTT solution (Cell Growth Assay; Merck) (5 mg/ml in PBS) was added to each well and incubated for 4 h at 37°C. The medium with MTT were removed, and 100 μl DMSO was added to dissolve formazan crystals at room temperature for 30 min. The optical density (OD) of each well was measured using an ELISA reader at 570 nm. The percentage of cell viability was calculated according to the following equation:

$$\begin{array}{l} \text{Cell}\;\text{viability}\left(\%\right)=\left[A_{570}\left(\text{sample}\right)/A_{570}\left(\text{control}\right)\right]\\ \;\times 100\% \end{array}$$

### Lactate dehydrogenase (LDH) assay

MCF-7 or MDA-MB-231 cells were cultured in 96-well plates (1×10^4^ cells/well). The plates were incubated overnight at 37°C and on the next day, 300 μl of culture media containing drug doses were added to each well, and the plates were incubated at 37°C in 5% CO_2_. 48 h later, 100 μl of medium from each well was carefully transferred to new plates. 100 μl of LDH substrate prepared according to the manufacturer’s procedure (Cytotoxicity Detection Kit, Roche Chemical Co.) was added to each well. After 20 min shaking at room temperature lactate dehydrogenase activity was determined by change in absorbance at 490 nm. All drug concentrations were tested at least in triplicate wells and the assays were repeated independently three times.

### TUNEL assay

TUNEL was carried out using an In Situ Cell Death Detection Kit, AP (Roche Diagnostics; Germany) according to the manufacturer’s instructions. Briefly, after 48 h treatment by 2 μM TAM, 200 μM tranilast or a combination two, the cells were fixed by adding 4% paraformaldehyde for 30 min. The fixed cells were washed in PBS, permeabilized with 0.1% Triton X-100 for 5 min on ice, and then incubated with 50 μl of terminal deoxynucleotidyl transferase end-labeling solution for 60 min at 37°C in a humidified chamber in the dark. Then, cells were counterstained in PI staining solution for 4 min at room temperature in the dark. The percentage of positively stained cells per total number of cells was counted under a fluorescence microscope at a magnification of 40**×** in five random fields and averaged.

### Acridine orange/ethidium bromide (AO/EB) staining

MCF-7 or MDA-MB-231 were plated in 24-well plates (10^5^ cells/well) and incubated overnight in a humidified 5% CO_2_ incubator at 37°C for 24 h. At that time, cells treated with 2 μM TAM, 200 μM tranilast or a combination two and incubated for 48 h. After that, cells harvested and stained with AO/EB dye mix (1 part of 100 μg/ml of AO and 1 part of 100 μg/ml of EB in PBS) on a clean microscope slide. The live, apoptotic and necrotic cells were observed under the fluorescent microscope at a magnification of 40**×**. Experiments were repeated for twice.

### DNA gel electrophoresis (DNA laddering)

The MCF-7 and MDA-MB-231 cells were grown in absence or presence of 2 μM TAM, 200 μM tranilast and combination of both for 48 h. Cellular DNA was then extracted from each cell line. The cells were lysed with 1% SDS in TE buffer and digested with proteinase K for 4 h at 56°C. The samples were extracted with phenol and chloroform and the DNA was precipitated with a 1/10 volume of 3 M sodium acetate and an equal volume of ethanol, pelleted at 13,000 × g and resuspended in TE buffer and 10 mg/ml of DNase-free RNase for 30 min at 37°C. Finally, extracted genomic DNAs was loaded and fractioned on 2% agarose gels; gels were stained with ethidium bromide and photographed. When DNA extracted from apoptotic cells is subjected to gel electrophoresis, a typical internucleosomal “ladder” of DNA fragments is produced.

### Real-time quantitative PCR (RQ-PCR) analysis

Total cellular RNAs were extracted from control or drug-treated cell pellets, 48 h after treatment with 2 μM TAM, 200 μM tranilast and combination both, using RNeasy Mini kit (Qiagen) in accordance with the manufacturer”s protocol. First strand cDNA was synthesized using QuantiTect Reverse Transcription Kit (Qiagen). Numbers of cDNA copies were calculated from the absorbance at 260 nm. Aliquots of the cDNA were combined with the QuantiFast® SYBER® Green PCR Master Mix from Qiagen and primers, and assayed in triplicate using a Rotor-Gene 6000 real-time RT-PCR. The primers were designed using the program BioEdit and BLAST searches (http://www.ncbi.nlm.nih.gov) carried out to confirm specificity of the selected nucleotide sequences and properties of primers are summarized in Table 1.

**Table 1:** Oligonucleotide primers used in real-time RT-PCR to amplify specific mRNAs together with sizes of amplified products

**Target mRNA**	**Sequence (5′ to 3′)**		**Product size (bp)**
GAPDH	Forward	actctggtaagtggatattgttgc	**162**
	Reverse	ggaagatggtgatgggatttc	
BAX	Forward	tgtttgctgatggcaacttc	**104**
	Reverse	gatcagctcgggcactttag	
BCL-2	Forward	gggatgcctttgtggaacta	**138**
	Reverse	ctcacttgtggcccaggtat	
TGF-β1	Forward	tgaaccggcctttcctgcttctcatg	**152**
	Reverse	gcggaagtcaatgtacagctgccgc	
TGF-β2	Forward	atgcggcctattgctttaga	**185**
	Reverse	taagctcaggaccctgctgt	
TGF-β3	Forward	cagggagaaaatccaggtca	**179**
	Reverse	cctggaaggcgtctaaccaag	
TGF-βR1	Forward	atcacctggccttggtcctgtgg	**140**
	Reverse	ggtcctcttcatttggcactcgatg	
TGF-βR2	Forward	gtctactccatggctctggt	**197**
	Reverse	atctggatgccctggtggtt	
TGF-βR3	Forward	tacagagagaggtcacact	**112**
	Reverse	gtcttcagatgccacaccag	

Analysis and fold differences were determined using the comparative 2 ^-ΔΔC^_T_ method. Quantitative values were obtained from the threshold cycle (C_T_) number at which the increase in fluorescent signal was associated with an exponential increase of PCR product. The C_T_ values from samples were plotted on the standard curve and the copy numbers was calculated with GAPDH as the internal control.

### Measurement of secretion of TGF-β1 by ELISA assay

The amount of TGF-β1 released into the culture media supernatant of breast cancer cells was quantitated using the Quantikine human TGF-β1 (R&D Systems; Minneapolis, MN; USA) according to manufacturer’s guidelines. After 1 × 10^5^ MCF-7 and MDA-MB-231 cells were plated onto 48-well plates, cells were treated with 2 μM TAM, 200 μM tranilast and a combination two for 48 h. Supernatant from conditioned medium from TAM and/or tranilast-treated cells were analyzed for TGF-β1 protein secretion by absorbance reading at 450 nm. Values are expressed as secreted TGF-β1 pg/ml/1 × 10^5^ cells.

### Wound-healing assay

The post-confluent MCF-7 and MDA-MB-231 cells were used in this experiment. Wounds with a constant diameter were made with a plastic tip (1 mm) and wounded monolayers were washed several times with medium to remove cell debris. For each well five areas along the length of the wound were chosen accidentally for photography under phase contrast microscope on an inverted microscope. After photography, the cells were incubated at 37°C in a humidified incubator containing 5% CO_2_ in medium containing 2% serum in the absence or 2 μM TAM, 200 μM tranilast and combination of both for 48 h and allowed to migrate. Photographs of the wound areas chosen on day 0 were again taken at 48 h. Experiments were carried out in triplicate.

### In vitro cell invasion assay

Cell invasion was determined using transwell chambers made from polycarbonate membrane filters with a pore size of 8 μm. Transwell filters in 6-well plates were coated with matrigel, hydrated for about 2 h in the tissue culture incubator with 500 μl serum free culture media in the bottom and 500 μl in the top of the chamber. After hydration of the matrigel, 5 × 10^5^ cells were plated in 500 μl serum-free medium on top of chamber, while 2 ml medium 10% FCS were placed in the lower chambers. TAM at 2 μM, tranilast at 200 μM or a combination two were added to the upper chambers. Cells without any drug were used as vehicle. After 48 h of incubation, the filters were removed, washed twice in PBS and fixed in 10% formalin for 15 min. After fixing at room temperature, the chambers are rinsed in PBS and stained with 0.2% crystal violet staining solution for 30 min. After washing the chambers by PBS, the cells at the top of the matrigel membrane were carefully removed by a number of cotton swabs. At this time all cells that remain are the ones that have invaded to the bottom side of the membrane. The number of cells was counted in 10 fields at random chosen using an inverted microscope at the 10× objective and plotted as the percentage of invading cells.

### Statistical analysis

Data were expressed as the mean ± standard error (SEM). Statistical analysis was conducted by using one-way analysis of the variance (ANOVA) and t-test. All statistical analyses were done using SPSS software 19.0 (SPSS, Inc., Chicago, IL, USA) and means were considered as statistically different for P < 0.05.

## Results

### Cytotoxic and anti-proliferative effects of TAM and/or tranilast on breast cancer cells

**Figure 1:**
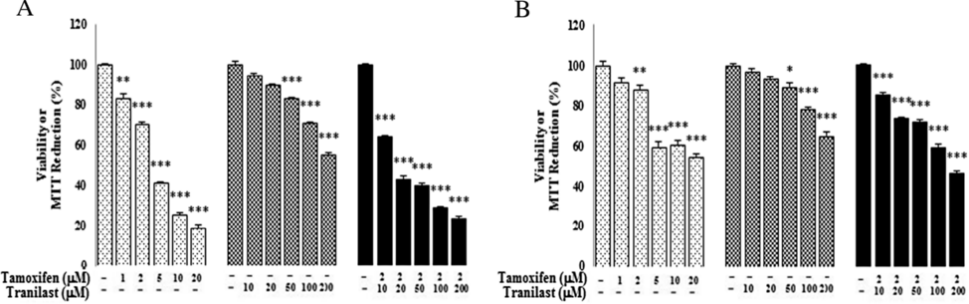
**The effects of TAM and/or tranilast on viability in MCF-7 (A) and MDA-MB-231 (B) cells.** Cells were treated with TAM, tranilast and combination both for 48 h. Control wells were treated with equivalent amount of media alone. Treatment with TAM and tranilast combined significantly decreased the viability compared with TAM or tranilast alone. The results showed the mean and SE from triplicated experiments. (*p < 0.05; **p < 0.01 ***p< 0.001 compared with control).

**Figure 2:**
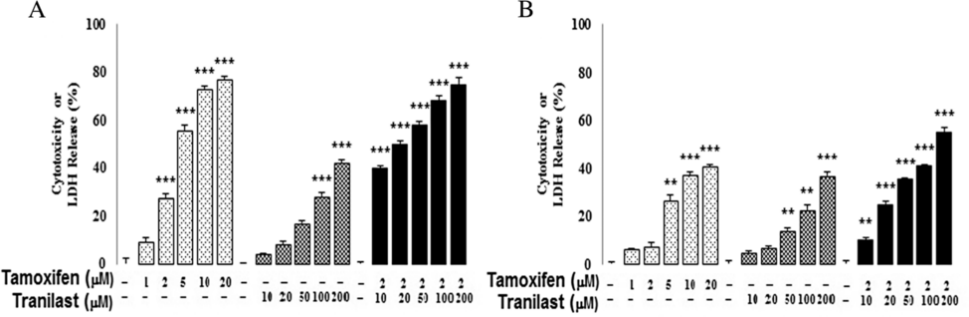
**TAM combined with tranilast additively inhibits survival of MCF-7 (A) and MDA-MB-231 (B) cells at 48 h.** Shown are the mean values of three experiments ± SE. p values were determined using one-way ANOVA (*p < 0.05, **p < 0.01, ***p < 0.001 compared with control).

The effects of TAM and tranilast alone or in combination on percent cell survival and proliferation was evaluated by MTT and LDH leakage assays. The results show that TAM and/or tranilast exhibits the anti-proliferative effect in a dose-dependent manner in both MCF-7 and MDA-MB-231 cell lines (Figures 1 and 2). The percentage of apoptotic cells in both cell lines after TAM and tranilast either alone or combined treatment was dramatically higher than in the untreated control cells. Especially, the percentage of apoptotic cells in the combined treatment was even higher than that in the treatment using the either agent alone (p < 0.001 for each comparison). The addition of tranilast to TAM caused a synergistic antiproliferative effect on dysplastic cells and an additive growth inhibition effect in both cell lines (Figures 1 and 2). Comparing the TAM and/or tranilast effect on growth between the two cell lines yields a significantly greater effect in the MCF-7 cell line than in MDA-MB-231 cell line (Figures 1 and 2).

### Apoptotic effects of TAM and/or tranilast on breast cancer cells

We investigated whether the combination of TAM and tranilast synergistically affected apoptosis of MCF-7 and MDA-MB-231 cells. To determine the effect of TAM, tranilast or combined both on apoptosis of MCF-7 and MDA-MB-231 cells, cells was treated with 2 μM TAM, 200 μM tranilast alone or combination two for 48 h.

For analyzing apoptosis, several assays were employed, including TUNEL assay, DNA fragmentation, AO/EB staining and to confirm apoptosis, we performed expression of bcl-2 and bax using real-time RT-PCR.

### TUNEL

**Figure 3:**
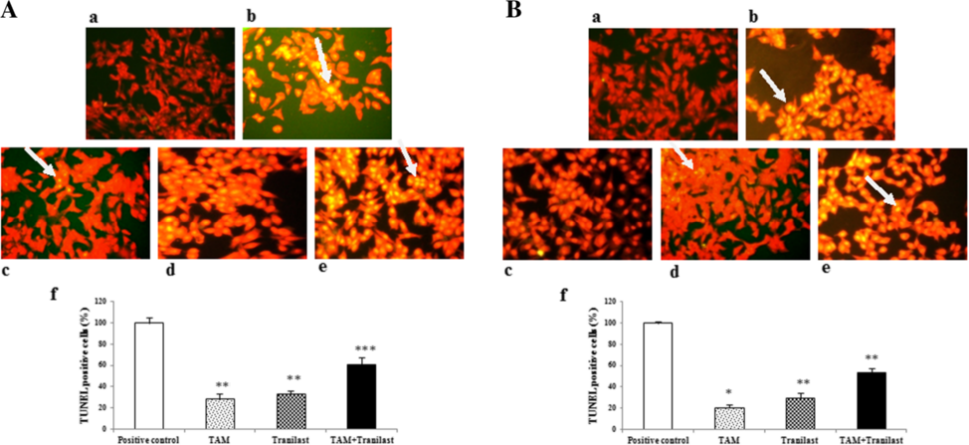
**Apoptotic potential of TAM and/or tranilast monitored by TUNEL staining in MCF-7 (A) and MDA-MB-231 (B) cells.** Phase micrographs of **a** negative control group; **b** positive control group; **c** 2 μM TAM; **d** 200 μM tranilast; **e** 2 μM TAM + 200 μM tranilast. **f** Columns mean percentage of apoptotic cells from three independent experiments performed in triplicate. White arrows indicate yellow-stained nuclei (TUNEL-positive cells). p values were determined using one-way-ANOVA (*p < 0.05 compared with control; **p < 0.01 compared with control). Scale bar: 10 μm.

The TUNEL reaction (TdT-mediated deoxy-uracil nick end labeling) is used for analyzing DNA fragmentation by labeling the 3′-OH ends of the DNA strand breaks. This method is based on the ability of terminal deoxynucleotidyl transferase (TdT) to attach a fluorescein-conjugated deoxy-uracil to the 3′-OH end of cut DNA [21]. Presented in Figure 2 TUNEL staining clearly displayed apoptotic cells in MCF-7 and MDA-MB-231 cells treated with TAM and tranilast alone or a combination two compared to untreated control cells. The numbers of apoptotic cells were quantitated and presented as percentages (Figure 3f). After treatment for 48 h, MCF-7 cells treated with TAM and tranilast alone as many as 29% and 33% of cells displayed TUNEL-positive staining, respectively, whereas 60% of the combination-treated cells were TUNEL-positive. As shown in Figure 3B, TAM and tranilast also induce a significant apoptosis in MDA-MB-231 cells (20% and 30%, respectively) after 48 h exposure. Under the same conditions, the percentage of TUNEL-positive MDA-MB-231 cells significantly increased with the combination of TAM and tranilast by 53%. As expected, the results show that in both MCF-7 and MDA-MB-231 cell lines, combination treatment resulted in higher levels of apoptosis than either of them alone (p < 0.01). In addition, TUNEL staining revealed an increased number of apoptotic cells in MCF-7 cells (Figure 3A) compared with MDA-MB-231 cells (Figure 3B).

### Acridine orange/ethidium bromide (AO/EB) staining

Cell death was divided into two types, necrosis and apoptosis. Necrosis causes inflammation while apoptosis does not. Induction of apoptosis in tumor cells has already been used as an important indicator to detect the ability of chemotherapeutic drugs to inhibit tumor growth [22].

Staining of apoptotic cells with fluorescent dyes such as AO and EB is considered the correct method for evaluating the changed nuclear morphology [23]. AO permeates all cells and the nuclei become green whereas EB is only taken up by cells that their cytoplasmic membrane integrity is lost, and their nuclei are stained red. EB also dominates over AO. Thus, live cells will show a normal green nucleus. Early apoptotic cells should give bright green nucleus with condensed or fragmented chromatin. Late apoptotic cells display condensed and fragmented orange chromatin and necrotic cells have a structurally normal orange nucleus [24].

**Figure 4:**
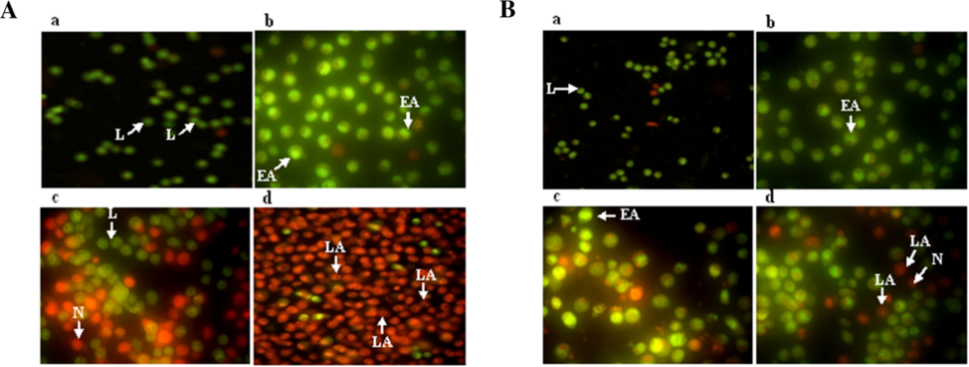
**MCF-7 (A) and MDA-MB-231 (B) cells were stained by AO/EB and observed under fluorescence microscope: a control group; b in the presence of 2 μM TAM; c in the presence of 200 μM tranilast; d in the presence of in combination both.** Green live cells show normal morphology; green early apoptotic cells show nuclear margination and chromatin condensation. Late orange apoptotic cells showed fragmented chromatin and apoptotic bodies. Scale bar: 10 μm. L = live cells, A = early apoptotic cells, LA = late apoptotic cells, N = necrotic cells.

The type of cell death induced by TAM, tranilast and combination of both studied by fluorescent staining for assessment of morphological changes. The Figure 4 exhibited morphological changes of apoptosis including cell shrinkage and chromatin condensation as compared to control cells. The live, apoptotic and necrotic and cells were monitored under the fluorescent microscope. From the results of Figure 4 we found that in MCF-7 cells, live cells were seen in the control group, both early and late apoptotic cells are seen in the presence of 2 μM TAM, while late apoptotic cells are obvious in the presence of 200 μM of tranilast and in the presence of combined treatment, the nearly all cells are late apoptotic cells (Figure 4A).

In MDA-MB-231 cells, live cells with normal morphology were seen in the control group, whereas early apoptotic cells occurred in the group with 2 μM TAM, early and late apoptotic cells were seen when 200 μM of tranilast and in the presence of combination both a number of cells in late stage, few cells also in early stage (Figure 4B). These morphological changes suggest that combination treatment significantly increased apoptosis in both MCF-7 and MDA-MB-231 cells.

### DNA fragmentation

This procedure is based on internucleosomal DNA cleavage, a characteristic biochemical hallmark of the apoptotic mode of cell death.

**Figure 5:**
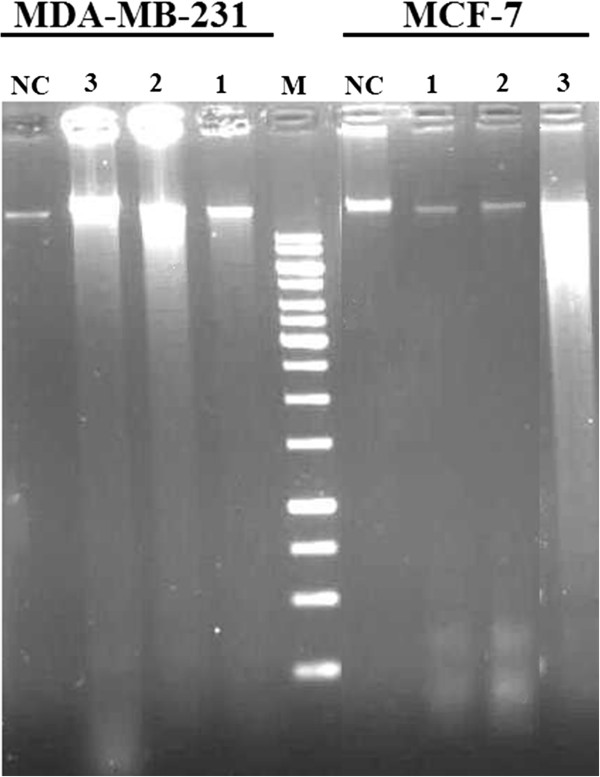
**DNA fragmentation in MCF-7 and MDA-MB-231 cells after treatment with 2 μM TAM, 200 μM tranilast and combination both.** Nuclear DNA was isolated from negative control and treated cells after 48 h and analyzed by agarose gel electrophoresis. M: DNA size marker; lane 1: 2 μM TAM; lane 2: 200 μM tranilast; lane 3: combination 2 μM TAM and 200 μM tranilast; NC: negative control (DNA from untreated cells).

Apoptosis of MCF-7 and MDA-MB-231 cells also detected by analysis of DNA fragmentation on agarose gel, a classical method of detecting the DNA ladders that accompany late apoptosis, *in vitro*. After treatment with 2 μM TAM, 200 μM tranilast and combination both for 48 h, the DNA extracted from cells was electrophoresed on 2% agarose gels. As shown in Figure 5, fragmented DNA was barely detectable. However, substantial amounts of low-molecular-weight DNA were present; indicating that either a small subset of cells had undergone internucleosomal DNA digestion or that only a fraction of each cell’s DNA had become fragmented. Although DNA fragmentation has been seen in many cell types and is generally considered the biochemical hallmark of apoptosis, it may be delayed, partial or absent in some cell types or experimental conditions. Therefore, it seems that treatment of MCF-7 and MDA-MB-231 cells with these doses not leads to significant DNA fragmentation (Figure 5).

Previous studies show also that treatment of epithelial cancer cell lines with a specific DNA-damaging agent will produce high molecular weight DNA fragmentation in the absence of nucleosomal laddering [25,26]. In Addition, some apoptosis studies fail to exhibit the DNA fragmentation pattern in the mammary carcinoma cells [21,27].

### Levels of bax and bcl-2 mRNA expression

To further investigate the apoptotic action of these two agents, we used quantitative real-time PCR to study the influence of them on bcl-2 and bax mRNA expression. In many human cancers, the anti-apoptotic bcl-2 proteins are overexpressed, or the pro-apoptotic proteins like bax, have reduced expression [28]. This results in resistance to a wide variety of cell death stimuli including chemotherapeutic drugs [29].

**Figure 6:**
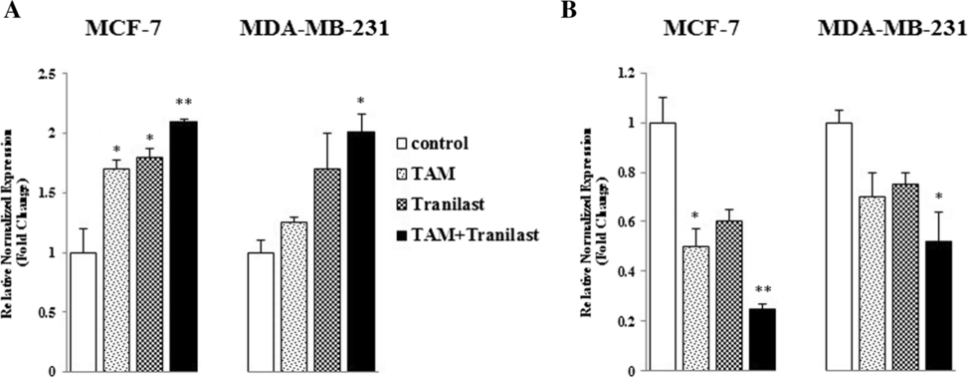
**Expression levels of apoptotic factors on MCF-7 cells after TAM and/or tranilast 48 h treatment. (A)** Bax (pro-apoptotic) and **(B)** Bcl-2 (anti-apoptotic). Bax/bcl-2 values > 1 mean the cell line enters apoptosis. p values were determined using one-way-ANOVA (*p < 0.05; **p < 0.01 compared with control).

Results of real-time quantitative PCR appeared to show down-regulation of bcl-2 and upregulation of bax expression at 48 hours treatment (Figure 6A, B). Expression of bcl-2 and bax was targets for TAM and tranilast as a single or combination and after 48 h exposure, a significant reduction of bcl-2 and induction of bax mRNA expression was observed. Bax to bcl-2 mRNA ratio was determined for MCF-7 cells: 3.4 in TAM treatment, 3.0 in tranilast treatment and 8.4 in combined group and for MDA-MB-231 cells: 1.7 in TAM treatment, 2.2 in tranilast treatment and 3.8 in combination. Hence, the ratio of pro-apoptotic to the anti-apoptotic was altered in favor of apoptosis (Figure 6). Thus, the results suggest that an up-regulation of bax and the corresponding down-regulation of bcl-2 mRNAs observed in this study may be one of the critical mechanisms through which TAM and/or tranilast induces apoptosis in breast cancer cells.

### Effects of TAM and/or tranilast treatment on mRNA level of TGF-β ligands and receptors in breast cancer cells

**Figure 7:**
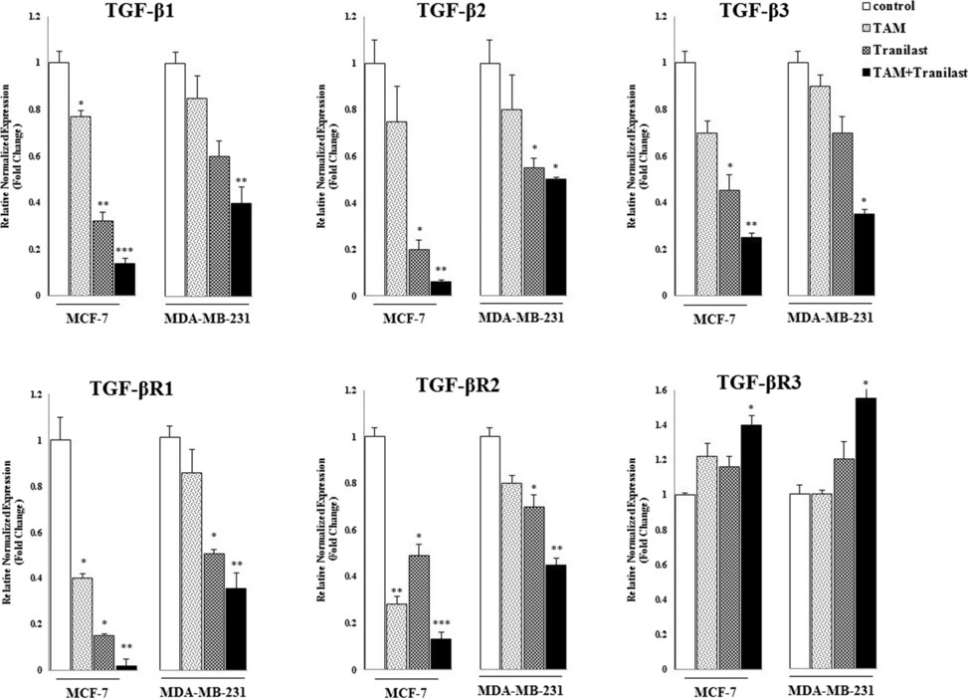
**TAM and tranilast regulate TGF-β ligands and receptors mRNA expression in MCF-7 and MDA-MB-231 cells.** Quantitative RT-PCR analysis in MCF-7 cells showed that TAM and tranilast as a single or in combination effectively decreased the expression of TGF-β1, 2, 3 and TβRI, II mRNA. However, MCF-7 cells are stimulated by TAM and/or tranilast to increase TβRIII expression slightly. The real-time RT-PCR results were normalized against the internal control GAPDH and are expressed as a percentage of control cells. All gene expression values were obtained in single experiments performed in triplicates. Different to control *p < 0.05, **p < 0.01, ***p < 0.001.

Exposure of cell cultures to TAM and tranilast either alone or in combination for 48 h decreased expression of TGF-β1, -β2, -β3 and TβRI, βRII mRNA. TGF-β1 mRNA levels were high but 48 h after TAM, tranilast or combined treatment they were diminished approximately a 30% (p < 0.05), 70% (p < 0.01) and 92% (p < 0.001) in MCF-7 cells and 15%, 40% and 60% (p < 0.01) in MDA-MB-231 cells (Figure 7). At the same time, mRNA expression of TGF-β2 in treatment with TAM or tranilast was down-regulated by 25% or 55% (p < 0.05) in MCF-7 and 15% or 45% (p < 0.05) in MDA-MB-231 respectively, while mRNA expression levels were decreased by approximately 10-fold in the presence of TAM plus tranilast (p < 0.01) in MCF-7 and 2-fold in MDA-MB-231 cells (p < 0.05) (Figure 7). Incubation of the cells for 48 h with TAM, tranilast or both down-regulate the mRNA encoding TGF-β3 by 40%, 60% (p < 0.05) and 80% in MCF-7 cells; and 10%, 30% and 65% in MDA-MB-231 cells respectively (Figure 7). Expression TβRI in TAM, tranilast or a combination two groups was decreased by approximately 2.5 (p < 0.05), 5 (p < 0.05) and 25-fold (p < 0.01) by MCF-7 cells, and 15%, 50% (p < 0.05) and 65% (p < 0.05) by MDA-MB-231 cells, respectively. Incubation of the cultured cell lines with TAM, tranilast or two drug decreased mRNA level encoding TβRII by 50% (p < 0.01), 55% (p < 0.05) and 87% (p < 0.001) in MCF-7 and 15%, 30% (p < 0.05) and 55% (p < 0.01) in MDA-MB-231 cells, respectively (Figure 7). However, Forty eight hours after TAM, tranilast or combined treatment the type III receptor mRNA levels were increased by about 20%, 50%, and 75% (p < 0.05) in MCF-7 and without difference, 20% and 55% (p < 0.05) in MDA-MB-231 cells, respectively compared with vehicle cells (Figure 7).

### Effect of TAM and/or tranilast on TGF-β1 secretion in MCF-7 and MDA-MB-231 breast cancer cells

**Figure 8:**
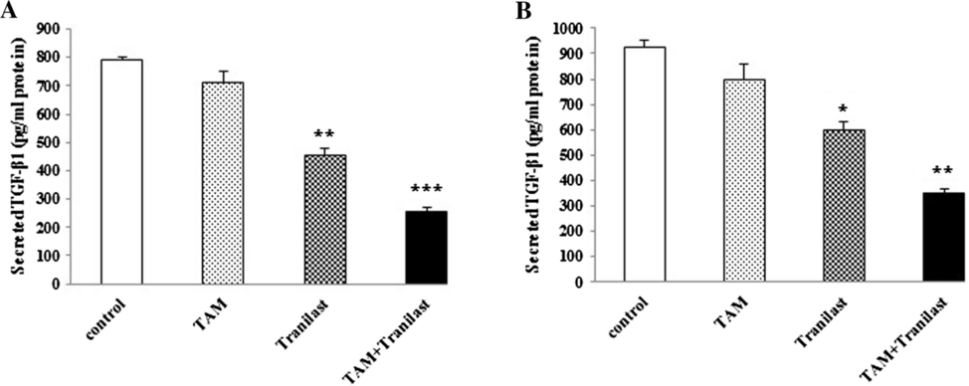
**Tranilast and TAM regulate the extracellular TGF-β1 protein secreted from cultured human breast cancer cells.** MCF-7 and MDA-MB-231 cells were cultured without drugs (control) or in the presence of 2 μM TAM, 200 μM tranilast or a combination two for 48 h. Results represent at least two independent experiments ± SEM. **(A)** TGF-β1 protein levels in medium from MCF-7 cells; **(B)** TGF-β1 protein levels in medium from MDA-MB-231 cells (*p < 0.05, **p < 0.01, ***p < 0.001).

To evaluate the effects of TAM and/or tranilast on TGF-β1 production from MCF-7 and MDA-MB-231 cells, we measured using ELISA kit secreted TGF-β1 protein level in the culture medium on cells treated with drugs alone or combination of both. We found that treating MCF-7 or MDA-MB-231 cells with TAM and tranilast as a single treatment for 48 h significantly decreased TGF-β1 secretion from breast cancer cell lines, compared to control (p=0.21 for TAM and p < 0.01 for tranilast in MCF-7; p=0.26 for TAM and p < 0.05 for tranilast in MDA-MB-231 cells). The minimum protein levels were observed as an effect of combination treatment (p < 0.01 in MCF-7 and p < 0.001 in MDA-MB-231 cells). These inhibitory effects also were higher in MCF-7 cells (Figure 8A) than in MDA-MB-231 cells (Figure 8B).

### Effects of TAM and/or tranilast on cell migration and invasion

**Figure 9:**
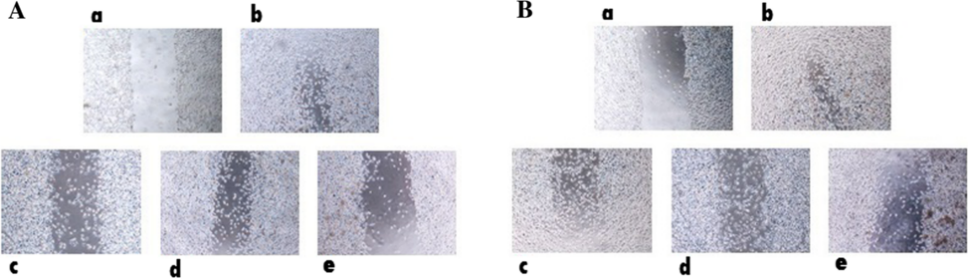
**Effect of TAM/or tranilast on invasion in MCF-7 (A) and MDA-MB-231 (B) cells.** Phase micrographs of cells were taken at 0 and 48 h after monolayer wounding. **a** control group in 0 day; **b** control group after 48 h; **c** MCF-7 cells treated with 2 μM TAM; **d** MCF-7 cells treated with 200 μM tranilast; **e** MCF-7 cells treated with combination of two. Results are the average of three independent experiments, in triplicates. Scale bar: 10 μm.

**Figure 10:**
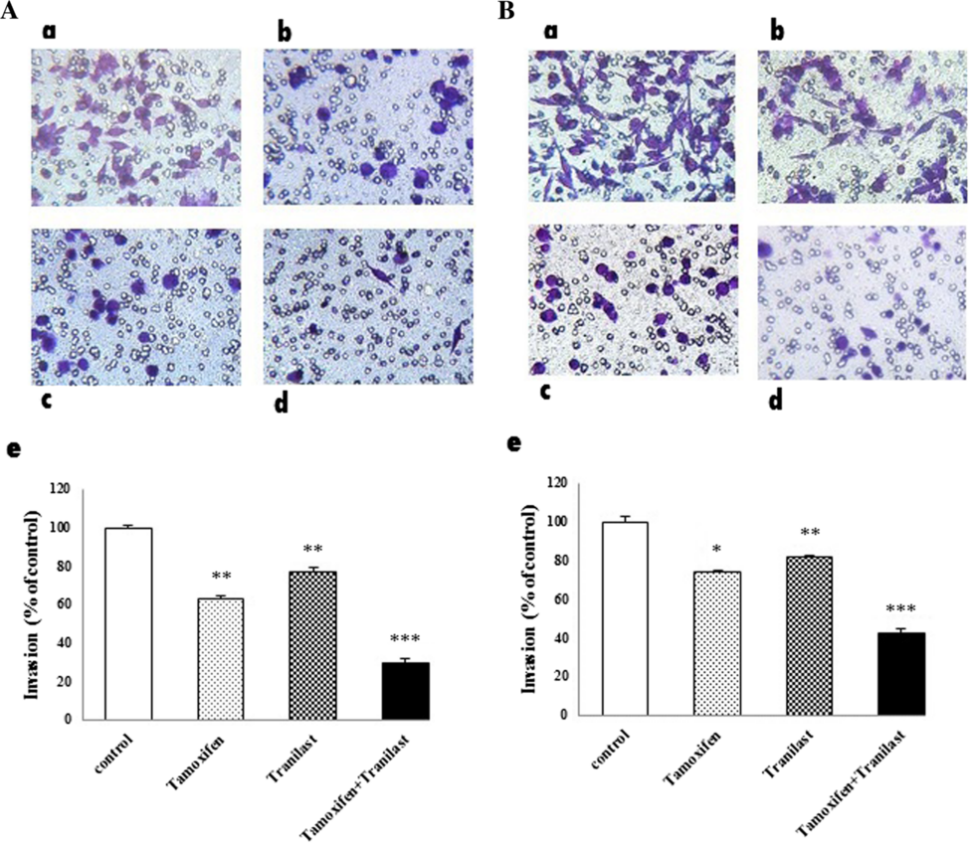
**Tranilast enhances anti-migratory activity of TAM in MCF-7 (A) and MDA-MB-231 (B) cells invasion *in vitro*.** Tamoxifen, tranilast and combination both inhibits MCF-7 cell invasion through matrigel. Phase micrograph of invading MCF-7 **(A)** and MDA-MB-231 **(B)** cells: **a** control group; **b** cells treated with 2 μM TAM; **c** cells treated with 200 μM tranilast; **d** cells treated with combination of two; **e** Quantification of cell invasion (*p < 0.05, **p < 0.01, ***p < 0.001). Scale bar: 10 μm.

To evaluate the effects of TAM and tranilast as a single or combined treatment on cell migration, we performed wound (scratch) and transwell invasion assays in MCF-7 and MDA-MB-231 cells. After 48 h treatment, cells in the control group efficiently spread into the wound area to such an extent that the wound boundary was not apparent, while only some cells in TAM or tranilast treated group spread forward in MCF-7 and MDA-MB-231 cells. The cell migration in combination group was lower than either drugs alone. (Figure 9A, B). In migration assay using a transwell system, migration was also decreased significantly with TAM or tranilast treatment. Combination TAM with tranilast decreased cell invasive ability of MCF-7 and MDA-MB-231 cells by 75% (p < 0.001) and 60% (p < 0.001), respectively (Figure 10A, B) compared with the control.

## Discussion

This study indicates that the effects of TAM with combination tranilast may be enhanced, which shows the mixture of TAM with tranilast produced a significant additive cytotoxic effect in both cell lines. Our data also demonstrated that TAM and tranilast inhibited MCF-7 and MDA-MB-231 cells proliferation by inducing apoptosis and the enhanced apoptosis may account for the synergistic inhibition of the combination treatment.

Disabling of apoptosis is a central event in tumorigenesis, and most chemotherapeutic drugs require functioning apoptotic pathways [2]. Estrogen results in a general up-regulation of genes regulating cell proliferation and survival and the down-regulation of genes with anti-proliferative or pro-apoptotic activity and the final resulting in growth stimulation and apoptosis suppression [30]. Therefore, antiestrogens are able to decrease cancer cell proliferation and induce cell death signaling pathways [31]. Consequently, tamoxifen treatment induces cell-cycle arrest leads to an accumulation of cancer cells in G0/G1 phase of the cell cycle [32] and induce apoptosis of breast cancer cells [33]. Morphological changes occur in apoptotic cells provide the most important means of diagnosing apoptosis, which the chromatin condenses and collapses into patches, followed by nuclear fragmentation and produce apoptotic bodies [34]. The Bcl-2 family of proteins, with pro- and anti-apoptotic members, regulates apoptosis during mammary gland development and mammary tumorigenesis [35]. It has been determined that both anti-apoptotic bcl-2 and pro-apoptotic bax contribute to mammary apoptosis [36] as well the bcl-2 gene is overexpressed in breast cancer cells [37].

In this work, synergistic effect of combination TAM and tranilast on induction apoptosis in breast cancer *in vitro* examined using some methods and changes in apoptotic cells evaluated. TAM and/or tranilast induced characteristic morphological modifications associated with apoptosis, including condensation of chromatin and DNA cleavage, as well expression of apoptosis regulators, bax and bcl-2 assessed and confirmed. We have demonstrated that the combination of TAM and tranilast resulted in a synergistic effect on both growth inhibition and apoptosis induction.

Studies have revealed that TAM is also effective in treatment of ER-negative tumors including breast [38]. The apoptosis induced by TAM is not reversible by addition of estrogens, telling that ER-independent induction of apoptosis could be a dominant mechanism of action in ER-negative breast tumors [39]. On the other side, inhibition of breast cancer growth by tamoxifen appears to be mediated by TGF-β signaling pathway [20]. Tamoxifen implements its effects both directly through the promotion of apoptosis and inhibition of mitosis, and indirectly through the TGF-β. It is found that changed expression of growth factors, among them TGF-β, is crucial for carcinogenesis [40]. TGF-β plays pivotal role in breast cancer. Some studies show that TGF-β is a potent inhibitor of primary mammary epithelial cells and breast cancer cell lines and reduced levels of TGF-β signaling are observed in several cancers [41,42]. Conversely, a large number of reports indicate that TGF-β turn into a promoter of progression in advanced tumor stages [43,44] by stimulation of angiogenesis, extracellular matrix degradation and metastasis [45]. Studies have shown a causal association between TGF-β and motility, invasiveness and metastasis [46] also survival and malignancy of human breast carcinoma cells [47]. Expression of TGF-β1, β-2, and β-3 mRNAs has been detected in human breast cancer cells [48]. Moreover, autocrine/paracrine TGF-β and its downstream Smad signaling play a survival role in breast cancer cells also Epithelial-Mesenchymal Transition (EMT) and lead to acquired tamoxifen resistance [49].

In this study tranilast with TAM down-regulated the expression of TGF-β1, β-2, and β-3 also TβRI and TβRII from breast cancer cells. TβRIII or betaglycan is a suppressor of breast cancer progression and that, when TβRIII expression is restored, invasion, angiogenesis, and metastasis is inhibited *in vivo*[50]. In this study, tranilast and TAM increased the expression of TβRIII slightly. Despite these uncertainties, it has become apparent that TGF-β gains a growth-promoting role and treatments that block TGF-β signaling have shown some efficacy in clinical trials [51].

Recently, there has been an increasing interest in evaluating combining chemotherapeutic drugs with other substances [52-55] for achieving better treatment with less toxicity in breast cancer. In this regard, we had chosen tranilast as an adjuvant to TAM in breast cancer therapy. Tranilast revealed no significant side effects even when administered for time-consuming periods and several reports showed that tranilast inhibits the proliferation of several cancer cell types including breast [56-59]. The inhibitory mechanisms have been elucidated as regards tranilast function, including its role in inhibiting and antagonizing the TGF-β pathway [60].

In the present study we show, tranilast as a single or in combination with TAM can regulate TGF-β isoforms and receptors gene expression and TGF-β1 protein secretion from human breast cancer cells. In addition, we demonstrate that tranilast and/or TAM inhibit migration and invasion of MCF-7 and MDA-MB-231 cells and these results could explain the beneficial effects of this combination in management of breast cancer. These results suggest that the additive effect between TAM and tranilast in inhibiting breast cancer may in part reflect the ability of both drugs to modulate and suppress TGF-β in breast cancer cells.

The anti-tumor effects observed here occurred at concentrations of tranilast that may well be achieved *in vivo.* If the results are confirmed *in vivo*, they may be significant clinically. Future researches on the detailed mechanisms of these using tranilast and tamoxifen will facilitate the understanding of the synergistic effects of these drugs on apoptosis as well TGF-β pathway.

## Conclusions

These results suggest that tamoxifen plus tranilast could be a promising combination therapy for future clinical trials in breast cancer patients. However further studies are also needed to investigate the expression of TGF-β pathway components in breast cancer contributes to the regulation of metastasis. Nonetheless, our study suggests that TGF-β pathway may be targeted for the inhibition of invasion in breast cancer cells. In a line, we believe that the present data may lead to new therapeutic options for breast cancer.

## Competing interests

The authors declare that they have no competing interests.

## Authors’ contributions

SD performed the experiments and drafted the manuscript; AGH designed research and performed the statistical analysis. Both authors read and approved the final draft of the manuscript.
